# Pathogenesis of spinal intramedullary lipomas: two case reports

**DOI:** 10.1186/s13256-023-04048-z

**Published:** 2023-07-25

**Authors:** Luis Miguel Moreno Gómez, Daniel García-Pérez, Pedro Juan González-León, Pablo M. Munarriz, Ana María Castaño-León

**Affiliations:** Neurosurgery Department, University Hospital “12 de Octubre”, Avda de Córdoba, s/n, 28041 Madrid, Spain

**Keywords:** Spine, Lipomas, Dysraphism, Recurrence, Malformation, Metabolism

## Abstract

**Background:**

Spinal lipomas not associated with dysraphism are rare and have an unknown natural history. In this report, we describe two cases; they showed recurrence during long-term follow-up, which makes us doubt a benign malformative etiology.

**Case reports:**

Two patients, a 19-year-old South American woman and a 14-year-old boy with spinal lipomas, underwent surgical resection. The lipomas were not associated with dysraphism and were located in the cervicothoracic and craniocervical junctions. In both cases, we decided to operate due to clinical progression; the former had a progressive natural course, and the latter experienced clinical worsening after recurrence from previous surgeries. The surgery took place with the assistance of neurophysiological monitoring and intraoperative ultrasound; a partial resection and medullary decompression were done, following the more recent recommendations.

**Discussion:**

The natural history of these lesions is currently unknown due to their rarity and the heterogeneity in the long-term follow-up of previously reported cases. Although previous reports describe good outcomes after surgical resection, long follow-ups, especially in young subjects, may show differences in these outcomes with progression and recurrence. We contribute to this last piece of evidence by describing two more cases of progression and recurrence.

**Lessons:**

Long-term close follow-up should be done in young subjects with spinal lipomas, as they are more prone to an aggressive course. Metabolism and hormonal changes may be behind this progression. Reoperation must be considered if neurological decline is detected.

## Background

Spinal lipomas represent 1% of all intramedullary lesions. They are frequently associated with spinal dysraphism, except for less than 1% of cases that cannot be associated with this malformation [1]. A cranial extension is very rare [2]. Their location makes clinical presentation vary, from asymptomatic cases to variable medullary symptoms and cranial nerve palsy. Treatment is controversial, especially in asymptomatic patients, because of their subpial intramedullary location and scarce evidence about their natural history [3].

There are few published reports of spinal lipomas without dysraphism, and it is even more infrequent to find cases with clinical worsening or radiological progression [3–5]. We aim to describe two subjects with aggressive progression during a long follow-up period and review the published literature.

## Case reports

### Case 1

**Fig. 1:**
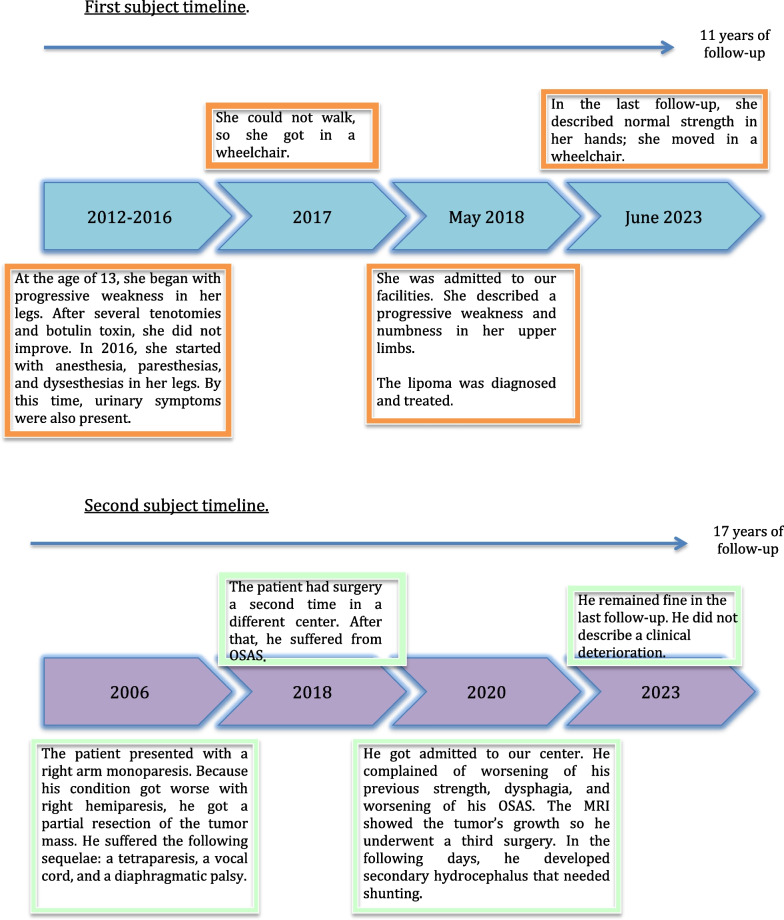
Subjects chronological timelines

A Hispanic 19-year-old South American woman was admitted to our center. She had a history of progressive spastic paraparesis for the last 5 years. She had been diagnosed with spastic diplegia and treated with several tenotomies without improvement. She had no other medical or surgical relevant history. She complained of worsening neurological status, describing a subacute onset of weakness in both hands (Fig. 1).

During the admission period, she presented with an acceptable general health condition. She had a heart rate of 95, with systolic and diastolic tension of 109 and 69, respectively. She did not present with infectious symptoms, and her cardiac, thoracic, and abdominal physical examinations did not show relevant information.

**Fig. 2:**
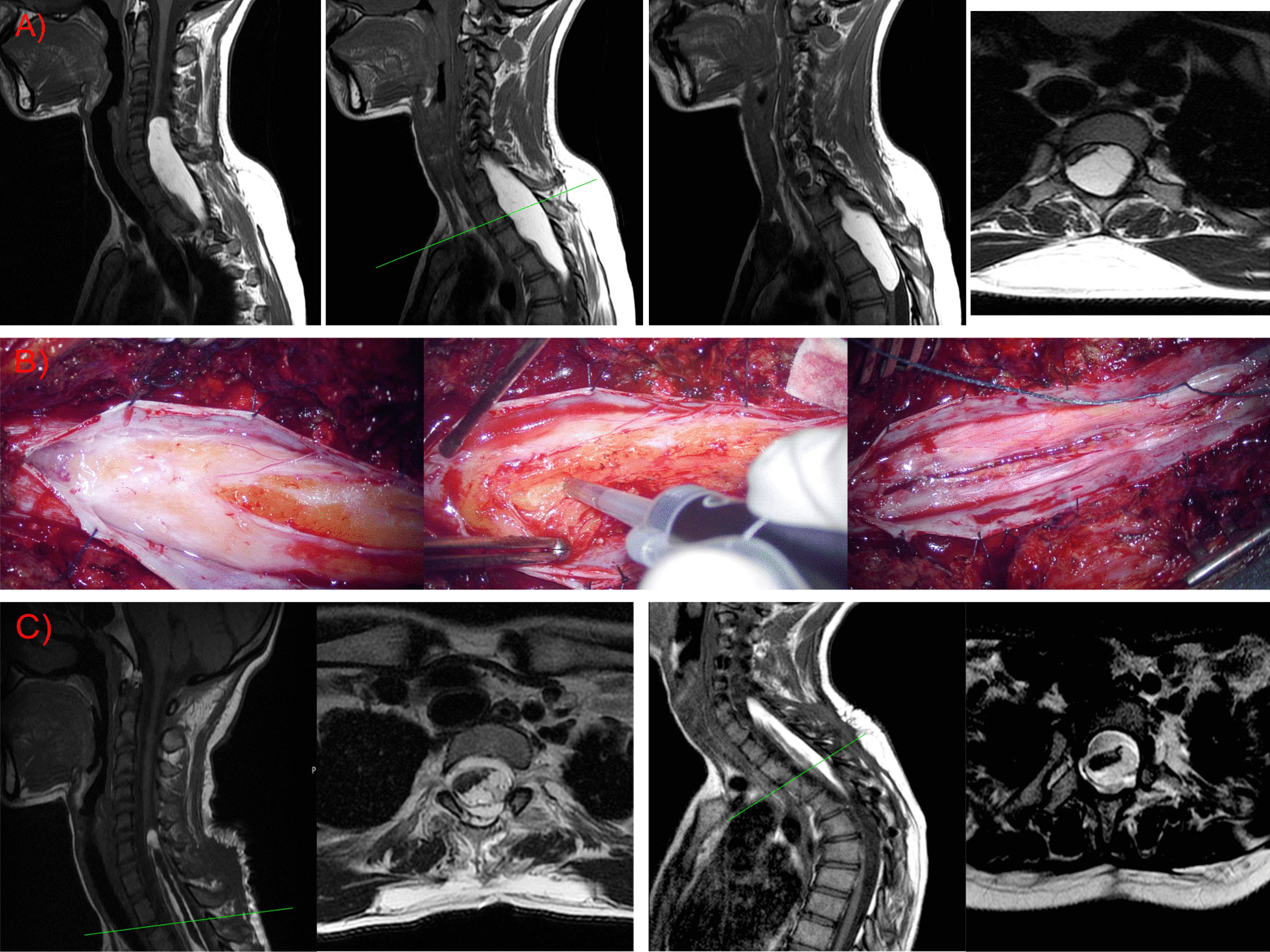
**A** Magnetic resonance images showing a spinal lipoma that went from the cervical region (C5) to the thoracic region (T5). Note the axial T2-weighted image (T2WI). This sequence confirmed the intramedullary subpial localization of the tumor. **B** Intraoperative images taken under the surgical microscope. Note the lipoma localization under a subpial coat, and the tumor remains anchored to the posterior part of the spinal cord. **C** Postoperative magnetic resonance imaging immediately after the surgical procedure and 2 years after surgery. No radiological progression is shown during this period, with the tumor remaining invariable. Green lines show the level where the sagittal magnetic resonance imaging is sliced to show the axial view

On the other hand, her neurologic examination revealed an incomplete cervical medullary cord syndrome with a C8 motor and sensitive level. She experienced weakness in her hands during flexion, abduction, and adduction movements (4/5). She had spastic paraplegia in both legs, with atrophic muscles, hyperactive myotatic reflexes, never-ending clonus, and bilateral extensor plantar response. She was unable to walk and used a wheelchair to move around. We found complete anesthesia below C8 level. Magnetic resonance imaging (MRI) showed an intramedullary lipoma that extended from C5 to T5, with flattening and anterior displacement of the medullary cord (Fig. 2A).

We indicated a surgical procedure due to the progressive course. Under neurophysiological monitoring and intraoperative echography guidance, a C5–D5 laminoplasty and a partial tumor resection were performed (Fig. 2B).

The postoperative course was uneventful, except for a cerebrospinal fluid (CSF) leak that required surgical repair. The patient underwent total recovery of the weakness of the upper limbs. After a 3-year follow-up, we have not detected clinical or radiological progression (Fig. 2C). She continues to have full strength in her hands (5/5); unfortunately, she has not recovered any strength in her legs and continues to depend on a wheelchair to move around. The sensory symptoms, although improved, continue in mild forms such as paresthesias and patch anesthesia. Finally, she continues with urinary and anal sphincter function problems.

### Case 2

A 14-year-old South American boy had a medical record of a previous cervical intramedullary lipoma with intracranial extension diagnosed when he was a newborn. At this time, he had only monoparesis of the upper right limb. During the follow-up, he presented clinical progression on two occasions, needing surgery in two different hospitals. Unfortunately, the surgical procedures never achieved a gross total resection. Due to the mass-effect progression and recurrent surgeries, the patient had sequelae consisting of dysphonia, dysphagia, obstructive sleep apnea syndrome (OSAS), and mild spastic tetraparesis. At our center, he complained about worsening of his previous dysphagia with frequent microaspiration-pneumonias and spasticity in his lower limbs (Fig. 1).

**Fig. 3:**
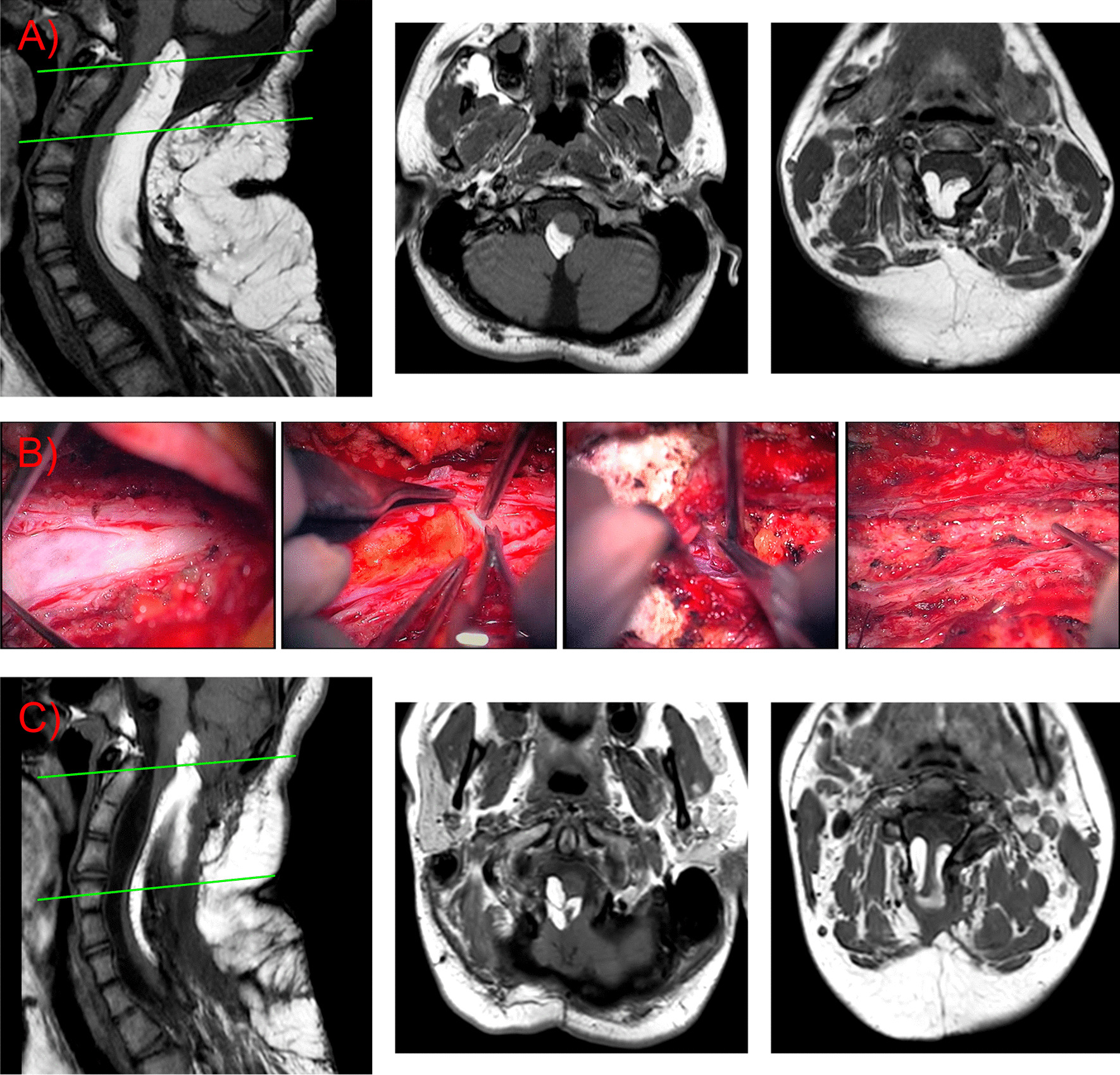
**A** Sagittal and axial Magnetic resonance images showing the cervicomedullary lipoma. Note the absence of laminas in the cervical vertebrae, a result of the previous surgical resections. **B** Intraoperative images. From left to right, we can see the arachnoid coat, the subpial lipoma, the cervicomedullary junction after lifting part of the lipoma, and finally, stimulation of residual tumor remains anchored to the posterior part of the medulla. **C** Postoperative MR images. The posterior lengthening of the medullary cord made resection of the tumor quite challenging. Green lines show the level where the sagittal magnetic resonance imaging is sliced to show the axial view

At admission, he looked deteriorated because of the several surgeries and sequelae experienced from them. Nevertheless, his vital signs were fine: heart rate of 58, systolic and diastolic tension of 102/63, and blood oxygen saturation of 99%. The neurologic examination showed low cranial nerve impairment with dysphonic speech and a right-side tongue deviation. Additionally, the subject experienced a general motor weakness in all group muscles (4+/5), except in the psoas and gastrocnemius where the strength was 4−/5. As in the other patient, signs of first motoneuron were identified, with hyperactive myotatic reflexes and clonus in the lower limbs. The patient’s gait was paretic, although he could stand and move alone. He did not experience sensory symptoms. MRI showed progression of the lipomatous tumor that extended from the bulb–medullary junction to T1, congruent with an intramedullary spinal lipoma with intracranial extension (Fig. 3A).

Recalling the first patient, we performed a surgical procedure owing to clinical progression. With the assistance of neurophysiological monitoring and intraoperative ultrasound, debulking of the lesion was done until direct stimulation of the residual mass evoked somatosensory responses (Fig. 3B). To conclude, an allogenic dural graft was sewn in a watertight fashion to the remaining dura.

Postoperative MRI showed improvement of the mass effect in the spinal cord (Fig. 3C) after partial removal of the lipoma. Unfortunately, this patient developed a deep surgical site infection due to a CSF leak. *Klebsiella pneumoniae* was the bacteria responsible for this deep surgical site infection. Fortunately, it was sensitive to the majority of antibiotic treatments. Initially, before bacteria identification, we used empiric antibiotic treatment with meropenem and linezolid; afterward, when knowing the pathogen susceptibility, we used specific antibiotic treatment with cefotaxime. The infection resolved after 20 days of antibiotics, and a debriding and cleaning new surgical procedure. After proper antibiotic treatment, we finally placed a ventriculoperitoneal shunt to treat secondary hydrocephalus. Ultimately, the patient went to a rehabilitation facility.

During these last 3 years, the patient has experienced an improvement in the dyspnea and OSAS symptoms that made him seek medical attention at our center. In addition, he has recovered strength in his lower limbs, helping him to walk better and improve stabilized. The ventriculoperitoneal shunt has not malfunctioned. The last MRI at our center shows tumoral remnants adherent to the medullary spinal cord without regrowth.

## Discussion

In this report, we describe two lipomas not associated with dysraphism. These are considered to account for less than 1% of all spinal lipomas. These lipomas have an unknown etiology, although disturbances in the normal embryogenesis of the neural tube could be responsible for them [3, 4, 6]. That would support the malformation or hamartomatous origin as the most plausible theory explaining their etiology [7, 8]. However, the progression in some of these surgically treated tumors gives rise to other theories, including the neoplasm feature [3].

The tumor’s symptoms depend on its location. The cervicothoracic junction and the thoracic spine are the most common regions [4, 6, 8–10]. Giuffrè [11] describes a progressive unilateral or bilateral motor weakness in lower limbs that rise to upper levels as the most frequent symptoms. Bhatoe *et al.* [4] report constant axial pain with dysesthesia. Also, sphincter disorders may be present. Exceptionally, cervical lipomas can extend into the intracranial compartment [12–14]. In this case, compression over the cranial nerves can be identified [12, 15].

Their unknown natural history makes treatment in asymptomatic patients with non-dysraphic lipomas controversial. However, surgery is the treatment of choice when the lipoma has a mass effect, especially in symptomatic patients [8, 9]. Generally, the consensus is to provide treatment as soon as possible: established loss of neurological function is related to a poor neurological outcome after surgery [4, 9, 16]. In our case, surgery was indicated as soon as possible because both patients had neurological progression.

Due to its etiology, resectioning these lesions is challenging because the cleavage plane with the adjacent medullary cord is indistinguishable. Complete resection attempts have led to disastrous complications, with partial resections with tumor debulking currently being accepted as the treatment of choice [4, 5, 7, 8, 17]. We performed partial resections with acceptable surgical risk, using the standard microsurgical techniques described for their resection [4, 5, 10, 16, 18]. In both, we observed arachnoid tissue surrounding the lipomas; this confirmed our initial suspicions about their subpial localization. We carefully resected the lipomatous tissue via a cavitron ultrasonic aspirator (CUSA^®^, Integra LifeSciences, Ireland), always under echography and neurophysiology guidance. We stopped the resection when a good decompression was achieved without intending to find a separation plane and leaving the most adherent remaining tumor to the medullary cord. The dura was closed in a watertight fashion with an allogenic dura graft and running suture; the objective was to leave a bigger space for the lipomatous spinal cord. Intraoperative echography and neurophysiology are imperative to achieve a safe mass resection, minimizing the postsurgical morbidity in these cases [4, 18].

The postsurgical outcome is uncertain, as some surgeons defend that resection improves or at least stops further decline of neurological function, while others state differently [4, 5, 10, 11, 19]. Unfortunately, both patients presented herein were already severely affected before, so the main objective was to detain neurological decay. In this regard, this purpose was, at the minimum, achieved. Patient 1 showed improved weakness in her upper limbs during the first postoperative weeks; patient 2 stopped his neurological decline. After 3 years of follow-up, they have continued to do well, and we have not detected a recurrence by MRI. In conclusion, based on our results, we suggest that spinal lipomas behave similarly to other intramedullary tumors, where being in an adequate preoperative neurological condition is critical to face this surgical process and postoperative course. This is supported by other authors [20, 21].

Unlike other non-dysraphic lipomas in literature, long-term follow-up is presented in both patients herein. That may be representative of their natural history. Currently, the natural history of these lesions is unknown owing to their rarity and the heterogeneity in the long-term follow-up of previously reported cases. Bhatoe *et al.* [4] published one of the broadest case series with long-term follow-up, not describing any clinical deterioration; they did not find an association between the resection grade and the rate of recurring cases. In concordance with this study, Giuffrè [11] observed similar outcomes. Contrarily, Lee *et al.* [5] and Fleming *et al.* [3] reported two cases of recurrence in children. We contribute to this evidence by describing two subjects that experienced clinical and radiological progression: the first case before treatment, and the second one after two surgical resections. The first subject began with symptoms 5 years before she arrived at our facilities. The tumor growth produced a neurological deterioration because of an incorrect diagnosis and treatment. After receiving surgical treatment at our hospital, the mass remained stable during the current 3 years of follow-up. On the other hand, the second patient had a lifelong history of neurological decline and reoperations. He was operated on for the third time when he was 14 years old, after experiencing a new neurological deterioration.

Although a malformative disorder is a principal theory behind non-dysraphic lipomas, aggressive natural history can occur; the subjects presented here show this, with a reason for disability. In these cases, a neoplasm etiology might be behind. Either way, it may be difficult to justify this course, but independently of their etiology (malformative or neoplasm), metabolic and hormonal changes may influence this tumor’s growth. This aligns with the recent findings by Endoh *et al.* [22] and Akyuz *et al.* [23]. In addition, this would explain why young people are more likely to suffer from this disease and why an aggressive course might be expected in some of these subjects. In our case, it would justify the progression experienced by the first patient before treatment because, at that time, the patient was going through puberty, and possibly, hormonal and metabolic changes supported the tumor growth. Besides, this would explain the numerous tumor progressions experienced by the second patient during his infancy and adolescence, development stages characterized by hormonal and metabolic changes. In addition, this would justify the recurrences described by Fleming *et al.* [3] and Lee *et al.* [5] in their case series, as most of the subjects who experienced recurrence were very young when first diagnosed, like our second subject. Although Endoh *et al.* [22] and Akyuz *et al.* [23] have reported cases where proper weight control helped diminish tumor mass, this is not always viable [3, 24]. In these cases, a tumoral etiology might be behind the lipoma or just insufficient control with medical management. Owing to these disparities, not all authors accept diet control treatment, reserving this approach for asymptomatic subjects or when surgery is not feasible [8, 22].

## Lessons

Spinal lipomas not associated with dysraphism can have an aggressive course in young subjects. Metabolism and hormonal changes may be behind this progression. For this reason, even after surgical decompression, we recommend long-term close follow-up, especially in young subjects, as recurrence of the mass effect and clinical deterioration can occur. Reoperation must be considered if neurological decline is detected.

## Data Availability

Data were acquired from the hospital database.

## Abbreviations

ASIA: American Spinal Cord Injury Association
MRI: Magnetic resonance imaging
CSF: Cerebrospinal fluid
CUSA^®^: Cavitron ultrasonic aspirator
